# In Situ Preparation of Metallic Copper Nanosheets/Carbon Paper Sensitive Electrodes for Low-Potential Electrochemical Detection of Nitrite

**DOI:** 10.3390/s24134247

**Published:** 2024-06-29

**Authors:** Xing Zhao, Guangfeng Zhou, Sitao Qin, Jingwen Zhang, Guanda Wang, Jie Gao, Hui Suo, Chun Zhao

**Affiliations:** State Key Laboratory of Integrated Optoelectronics, College of Electronic Science and Engineering, Jilin University, Changchun 130012, China; zhaoxing1920@mails.jlu.edu.cn (X.Z.); zhougf22@mails.jlu.edu.cn (G.Z.); tanst1921@mails.jlu.edu.cn (S.Q.); zjw22@mails.jlu.edu.cn (J.Z.); gdwang19@mails.jlu.edu.cn (G.W.); gao1229jie@163.com (J.G.); suohui@jlu.edu.cn (H.S.)

**Keywords:** electrochemical sensor, catalytic reduction of nitrite, low detection potential, one-step electrodeposition method, Cu/CP

## Abstract

In the realm of electrochemical nitrite detection, the potent oxidizing nature of nitrite typically necessitates operation at high detection potentials. However, this study introduces a novel approach to address this challenge by developing a highly sensitive electrochemical sensor with a low reduction detection potential. Specifically, a copper metal nanosheet/carbon paper sensitive electrode (Cu/CP) was fabricated using a one-step electrodeposition method, leveraging the catalytic reduction properties of copper’s high occupancy d-orbital. The Cu/CP sensor exhibited remarkable performance in nitrite detection, featuring a low detection potential of −0.05 V vs. Hg/HgO, a wide linear range of 10~1000 μM, an impressive detection limit of 0.079 μM (S/N = 3), and a high sensitivity of 2140 μA mM^−1^cm^−2^. These findings underscore the efficacy of electrochemical nitrite detection through catalytic reduction as a means to reduce the operational voltage of the sensor. By showcasing the successful implementation of this strategy, this work sets a valuable precedent for the advancement of electrochemical low-potential nitrite detection methodologies.

## 1. Introduction

Nitrite is an intermediate product of the nitrogen cycle in the natural environment [1]. The excessive use of nitrogen fertilizers in agriculture and the indiscriminate discharge of industrial wastewater have led to serious nitrite pollution of underground water resources [2]. A high nitrite content in drinking water can react with protein breakdown in the human body, forming secondary amine compounds that further produce highly carcinogenic n-nitrosamines, posing irreversible harm to human health [3]. Therefore, developing an accurate, rapid, simple, and efficient nitrite detection method is crucial for protecting the ecological environment and other aspects. Common methods for nitrite detection include spectrophotometry [4], chemiluminescence [5], chromatography [6], capillary electrophoresis [7], and electrochemical methods. Electrochemical nitrite sensors have garnered attention due to their advantages such as ease of use, environmental friendliness, compact size, and suitability for on-site detection [8].

In electrochemical nitrite detection, the strong oxidizing nature of nitrite necessitates a high electrochemical oxidation detection potential, requiring a substantial bias voltage for the electrochemical sensor during detection [9]. Noble metals like Au, Pt, and Pd, known for their catalytic abilities, are frequently used in constructing electrochemical nitrite sensors [10]. For instance, Hu et al. developed a nitrite electrochemical sensor based on a unique flower-like Pd-ZnO nanostructure, which significantly reduced the nitrite oxidation detection potential to +0.78 V (vs. SCE) and exhibited a wide linear concentration range, low detection limit, and high sensitivity [11]. Despite the remarkable performance of precious metal-based nanomaterials in lowering detection potential [12], challenges remain in significantly reducing sensor detection potential in electrochemical nitrite detection based on the catalytic oxidation of nitrite [13].

It is important to note that nitrite exhibits not only strong oxidizing properties but also strong reducing properties [14]. Therefore, considering the catalytic reduction of nitrite, utilizing the reduction potential for nitrite detection can be an effective approach to lowering the operating voltage of the sensor [15,16]. For instance, Wang et al. developed a novel polyaniline nanofiber (PANI-NF) electrochemical sensor that catalyzed the conversion of nitrite ions to nitric oxide at a low potential difference, enabling the reduction detection of nitrite ions with a detection limit of 0.05 μM [17]. Similarly, Deng et al. created a polythionine/carbon nanotube/GCE-sensitive electrode through the electro-polymerization of thionine on the surface of carbon nanotube-modified glassy carbon, achieving high sensitivity and enhanced catalytic reduction currents with a reduced nitrite detection potential [18]. Therefore, it is feasible to design an electrochemical nitrite sensor with high sensitivity and low detection potential based on the electrocatalytic reduction mechanism of nitrite. Approaching the preparation of electrochemical nitrite sensors from the perspective of catalyzed nitrite reduction allows for efficient nitrite detection while achieving a reduced detection potential.

Notably, the d-orbital energy levels of certain highly occupied d-orbital metals such as Cu and Ag align well with the lowest unoccupied molecular orbital of NO_2_^−^, facilitating electron transfer on the metal surface. When a current is applied to the metal surface, H_2_O is reduced to stable adsorbed hydrogen (H_ads_), which in turn can reduce adsorbed NO_2_^−^ on the metal surface [19]. Copper, being an affordable transition metal with strong nitrite-catalyzed reduction ability, is a promising candidate for nitrite sensors [20]. Furthermore, the choice of the sensitive electrode substrate plays a crucial role in the development of high-performance nitrite sensors. Carbon paper (CP) stands out as a lightweight, chemically stable, and electrically conductive three-dimensional carbon substrate [21]. Therefore, growing highly occupied d-orbital metallic copper with excellent catalytic reduction ability on a carbon paper substrate proves to be an effective method for crafting binder-free electrochemical nitrite sensors with high sensitivity and low detection potential.

**Figure 1:**
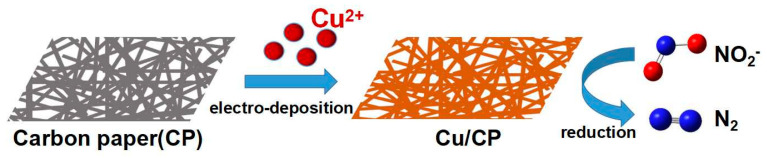
Preparation of Cu/CP−sensitive electrodes and catalytic reduction of nitrite.

In conclusion, as illustrated in Figure 1, this study introduces the preparation of copper metal nanosheets/carbon paper (Cu/CP) self-supported sensitive electrodes through a straightforward one-step electrodeposition method and evaluates the nitrite detection performance of these sensitive electrodes. The results demonstrate that the Cu/CP-sensitive electrode exhibits a broad linear range, high sensitivity, low detection limit, low detection potential, good anti-interference properties, and stability for nitrite detection. Moreover, the application of the self-supported Cu/CP-sensitive electrode in detecting nitrite in actual drinking water validates its practicality. This strategy serves as a valuable reference for achieving electrochemical low-potential nitrite detection.

## 2. Materials and Methods

### 2.1. Chemical Reagent

All chemical regents were of an analytical grade and were used without further purification. Copper chloride dihydrate (CuCl_2_·2H_2_O), ammonium oxalate monohydrate ((NH_4_)_2_C_2_O_4_·2H_2_O), polyethylene glycol 4000 (PEG4000), sodium nitrite (NaNO_2_), potassium chloride (KCl), and potassium ferricyanide (K_3_[Fe(CN)_6_]) were purchased from Beijing Chemical Works Co., Ltd. (Beijing, China). Ammonium chloride (NH_4_Cl), potassium carbonate (K_2_CO_3_), magnesium sulfate heptahydrate (MgSO_4_·7H_2_O), sodium nitrate (NaNO_3_), ferric chloride hexahydrate (FeCl_3_·6H_2_O) and potassium hydroxide (KOH) were obtained from Sinopharm Chemical Reagent Co., Ltd. (Shanghai, China). Hydrochloric acid (HCl), glucose (C_6_H_12_O_6_) and urea (CO(NH_2_)_2_) were acquired from Yongda Chemical Regent Co., Ltd. (Tianjin, China). Carbon paper (CP) was purchased from Carbon Enger Technology Co., Ltd. (Taiwan, China).

### 2.2. Pre-Treatment of Carbon Paper

The CP was cut into small rectangular pieces (2 × 1 cm^2^), and later sonicated with toluene, acetone, ethanol, 1 M HCl, and deionized water sequentially for 10 min each. Afterwards, the carbon paper was rinsed using deionized water to make it pH-neutral and stored in deionized water for spare use.

### 2.3. Preparation of Cu/CP-Sensitive Electrodes

The sensitive electrodes were prepared using a simple one-step constant potential deposition method. First, 5 mM CuCl_2_·2H_2_O, 10 mM (NH_4_)_2_C_2_O_4_·2H_2_O, and 0.005 g of PEG4000 were dissolved in 50 mL of 0.1 M HCl with continuous stirring to obtain a uniform and stable electrodeposition solution.

After that, constant potential deposition was carried out based on the three-electrode system, with the pretreated CP as the working electrode, Ag/AgCl electrode as the reference electrode, and 1.5 cm × 1.5 cm Pt sheet as the counter electrode. The constant potential deposition was applied for 1200 s at −0.65 V vs. Ag/AgCl. Finally, the Cu/CP was rinsed several times with deionized water and dried for 1 h at 60 °C to obtain the Cu/CP-sensitive electrode.

### 2.4. Characterization and Electrochemical Testing

The surface morphology and structure of the prepared electrodes were investigated using field emission scanning electron microscopy (SEM, JEOL-JEM-6700F, JEOL, Ltd., Tokyo, Japan). The crystal structure of the prepared electrodes was characterized using X-ray diffraction spectroscopy (XRD, D8 ADVANCE, Cu Kα source (λ = 1.54 Å)). The surface chemistry and valence states of the prepared electrodes were analyzed using X-ray photoelectron spectroscopy (XPS, ESCALAB-250).

The electrochemical characterization and testing were carried out on an electrochemical workstation (CHI 760D, Chenhua, Shanghai, China) using a typical three-electrode system, with the electrolyte being a 0.1 M KOH solution, the working electrodes being a bare CP electrode and a Cu/CP-sensitive electrode, and the counter and reference electrodes being a Pt sheet (1.5 cm × 1.5 cm) and a Hg/HgO electrode, respectively.

## 3. Results and Discussion

### 3.1. Characterization of Cu/CP-Sensitive Electrodes

**Figure 2:**
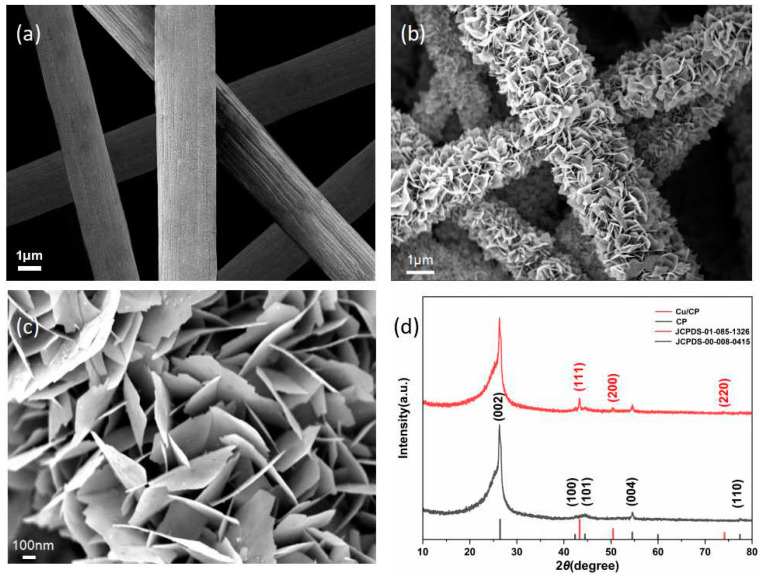
(**a**) SEM images of CP electrodes at low magnification. (**b**) SEM images of Cu/CP-sensitive electrodes at low magnification and (**c**) high magnification. (**d**) XRD spectra of Cu/CP-sensitive electrodes.

The surface morphology and microstructure of the Cu/CP-sensitive electrodes and CP electrodes were examined using scanning electron microscopy (SEM), and the results are shown in Figure 2a–c. Figure 2a shows the SEM images of the carbon paper, which consists of many interlaced carbon fibers with a smooth surface and an average diameter of about 2 μm. Figure 2b,c shows the SEM images of the Cu/CP-sensitive electrodes, and from the low magnification images, it can be observed that the metallic copper grows densely on the surface of each carbon fiber. A closer look under high magnification reveals that the metallic copper exists in the form of nanosheets with a thickness of about 10 nm. The growth of copper nanosheets on the surface of the carbon paper fibers increases the contact area between the sensitive electrode and the analyte, which improves the performance of the sensitive electrode for the detection of nitrite.

Furthermore, the crystal structures of the Cu/CP-sensitive electrode and the bare CP electrode were analyzed utilizing X-ray diffraction spectroscopy (XRD), as illustrated in Figure 2d. In the case of the bare CP electrode, diffraction peaks were observed at 26.3°, 42.3°, 44.5°, 54.5°, and 77.3°, corresponding to the (002), (100), (101), (004), and (110) crystal faces of graphitic carbon (JCPDS-00-008-0415) [22], respectively. On the contrary, the Cu/CP-sensitive electrode exhibited additional diffraction peaks at 43.3°, 50.3°, and 73.9°, aligning with the (111), (200), and (220) crystal faces of Cu (JCPDS-01-085-1326) [23], respectively. Notably, no diffraction peaks associated with other metallic copper-related compounds were detected, indicating the pristine Cu metal single phase nature of the prepared sensitive material.

**Figure 3:**
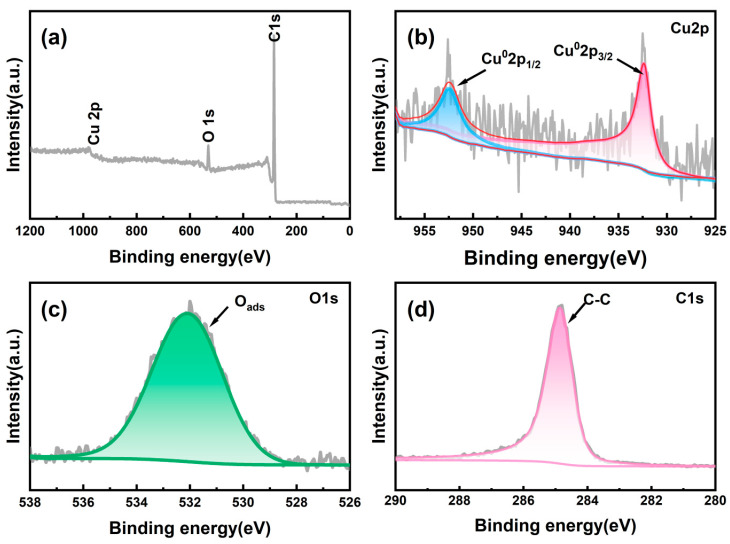
XPS spectra of Cu/CP electrodes: (**a**) measured spectrum, (**b**) Cu 2p, (**c**) O1s, (**d**) C1s.

The elemental composition and valence states of the Cu/CP-sensitive electrode were investigated through X-ray photoelectron spectroscopy (XPS), with the findings presented in Figure 3a–d. The full spectrum displayed in Figure 3a reveals only characteristic peaks corresponding to the C, Cu, and O elements, with the absence of characteristic peaks from other impurity elements, underscoring the high purity of the electrode-sensitive material. In the C 1s energy spectrum (Figure 3d), a solitary peak attributed to the C-C bond at 284.8 eV was identified, indicative of the carbon paper composition.

Analysis of the Cu 2p energy spectrum (Figure 3b) unveiled two peaks centered at 932.4 eV and 952.4 eV, corresponding to Cu 2p_3/2_ and Cu 2p_1/2_, respectively, signifying the presence of copper in the 0 valence state within the prepared electrodes [24]. Moving on to the O 1s energy spectrum (Figure 3c), the peak located at 532.1 eV was associated with surface adsorbed oxygen (O_ads_), such as O^2−^, O^−^, and -OH groups [15]. These results align with the XRD outcomes, further validating the successful fabrication of the self-supported Cu/CP-sensitive electrodes.

### 3.2. Electrochemical Behaviors of Cu/CP

**Figure 4:**
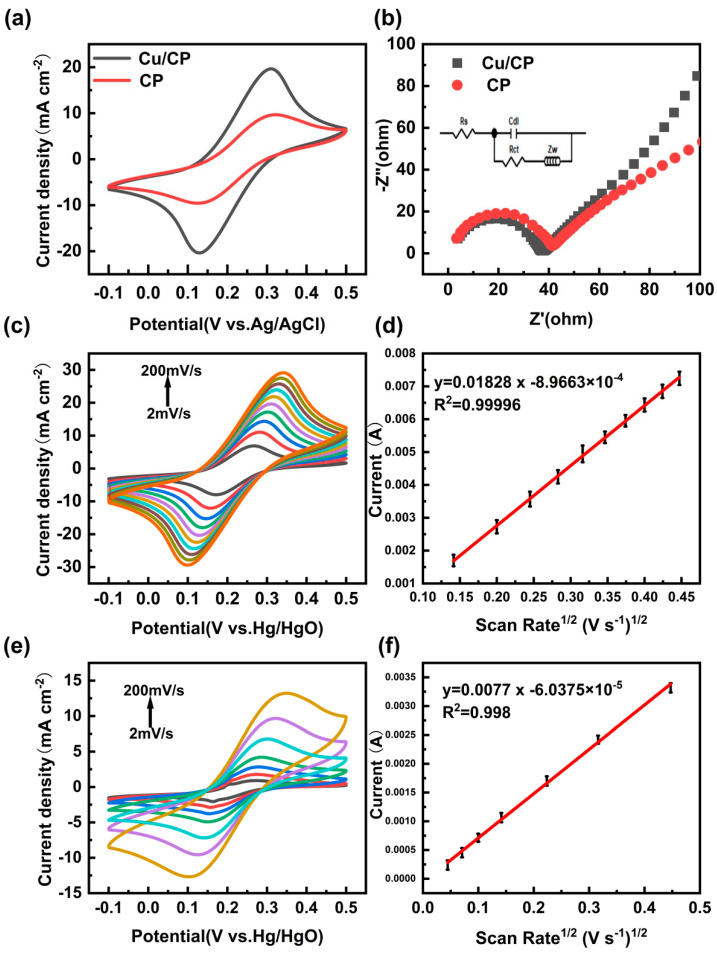
(**a**) CV curve and (**b**) EIS spectra of Cu/CP − electrodes in 0.1 M KCl solution containing 5.0 mM [Fe(CN)_6_]^3−/4−^. (**c**) CV curves of Cu/CP electrodes in 0.1 M KCl solution containing 5.0 mM [Fe(CN)_6_]^3−/4−^ at different scan rates (20, 40, 60, 80, 100, 120, 140, 160, 180, 200 mVs^−1^). (**d**) Linear relationship between the peak oxidation current of the Cu/CP electrodes and the square root of the scan rate. (**e**) CV curves of bare CP electrodes in 0.1 M KCl solution containing 5.0 mM [Fe(CN)_6_]^3−/4−^ at different scan rates (2, 5, 10, 20, 50, 100, 200 mVs^−1^). (**f**) Linear relationship between oxidation peak current and square root of scan rate for bare CP electrodes.

The comparison of electron transfer kinetics and interfacial electrochemical properties between the Cu/CP-sensitive electrode and the bare CP electrode was conducted, with the results depicted in Figure 4a,b. In Figure 4a, the cyclic voltammetry (CV) curves of both electrodes in 0.1 M KCl solution containing 5 mM potassium ferricyanide exhibited redox peaks. Notably, the peak current density of the Cu/CP-sensitive electrode was twofold higher than that of the bare CP, indicating a larger active surface area and enhanced conductivity attributed to the incorporation of Cu nanosheets on the CP surface [25].

Figure 4b showcases the Nyquist plots of the Cu/CP and bare CP electrodes, with the corresponding equivalent circuit diagrams provided in the inset. The Nyquist plot typically consists of a semicircular portion at high frequencies and a linear portion at low frequencies, where the charge transfer resistance (R_ct_) of the electrode is reflected by the semicircular diameter [26]. A smaller semicircular diameter signifies a reduced R_ct_, which is advantageous for promoting electrochemical reactions [27]. Analysis of Figure 4b reveals that the semicircular diameter of the Nyquist plot for the Cu/CP-sensitive electrode is smaller compared to that of the bare CP electrode, indicating a lower charge transfer resistance (R_ct_). Fitting results yielded R_ct_ values of 34.17 Ω and 37.19 Ω for the Cu/CP-sensitive electrode and the bare CP electrode, respectively, underscoring the decreased charge transfer impedance following the introduction of Cu nanosheets on the CP surface, thereby facilitating electrochemical reactions at the interface.

To assess the electrochemically active specific surface area (ECSA) for both electrodes, CV curves were obtained for the Cu/CP-sensitive electrode and the bare CP electrode in 0.1 M KCl solution containing 5 mM potassium ferrocyanide at varying sweep rates, as illustrated in Figure 4c,d. The oxidation peak current (I_pa_) of the Cu/CP-sensitive electrode exhibited a linear increase with the square root of the sweep rate. The ECSA was calculated using the Randles-Sevcik equation, as defined in Equation (1) [28].

I_pa_ = (2.69 × 10^5^)n^3/2^ACD^1/2^ν^1/2^ (1)

In Equation (1), Ipa, n, A, C, D, and ν represent the oxidation peak current (A), the number of electrons transferred (n was 3), the electrochemical active surface area of the investigated electrode (cm^2^), the concentration of the probe molecules (5 × 10^−6^ mol cm^−3^), the diffusion coefficient (7.60 × 10^−6^ cm^2^ s^−1^), and the scan rate (V/s), respectively. Resulting in ECSA values of 3.436 cm^2^ and 1.447 cm^2^ for the Cu/CP-sensitive electrode and bare CP electrode, respectively (Figure 4e,f). These findings demonstrate that the incorporation of metallic Cu nanosheets on the carbon paper surface enhanced the electrode’s properties, leading to low charge transfer resistance, high conductivity, a large electrochemically active specific surface area, increased electrochemically active sites, a rapid electron transfer rate, and superior electrocatalytic activity. These enhancements facilitated the electrocatalytic reduction reaction of nitrite, thereby improving the detection performance of the sensitive electrode [22].

### 3.3. Electrochemical Determination of Nitrite

**Figure 5:**
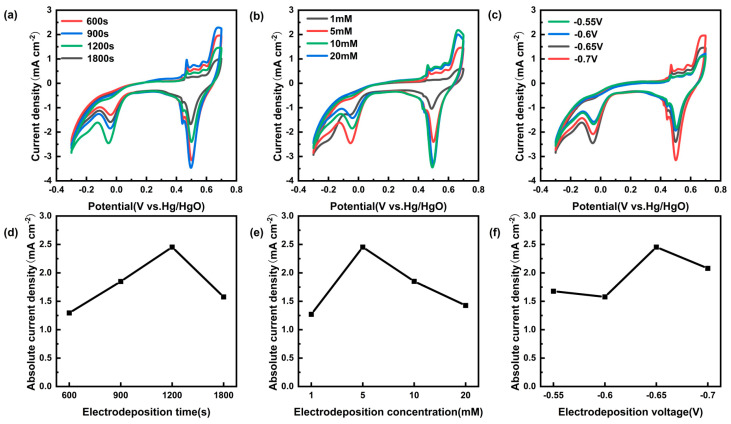
(**a**) CV curves of Cu/CP−sensitive electrodes prepared at different electrodeposition times with 1 mM nitrite added to 0.1 M KOH electrolyte. (**b**) CV curves of Cu/CP-sensitive electrodes prepared under different electrodeposition concentrations with the addition of 1 mM nitrite in 0.1 M KOH electrolyte. (**c**) CV curves of Cu/CP-sensitive electrodes prepared at different electrodeposition potentials with the addition of 1 mM nitrite in 0.1 M KOH electrolyte. (**d**) Comparative line graphs of reduced peak currents of CV curves of Cu/CP-sensitive electrodes prepared at different deposition times. (**e**) Comparison of the CV curves of Cu/CP-sensitive electrodes prepared at different electrodeposition concentrations in terms of the reduced peak currents of the line graphs. (**f**) Comparison of the CV curves of the Cu/CP-sensitive electrodes prepared at different electrodeposition potentials with the reduction peak current fold plot.

Optimization of the preparation conditions for Cu/CP-sensitive electrodes through electrodeposition resulted in the identification of key parameters, as depicted in Figure 5. The optimal conditions included a deposition solution containing 5 mM Cu^2+^, a deposition potential of −0.65 V (vs. Ag/AgCl), and a deposition time of 1200 s. Subsequently, Cu/CP-sensitive electrodes prepared under these optimized conditions were selected for further electrochemical assessments.

**Figure 6:**
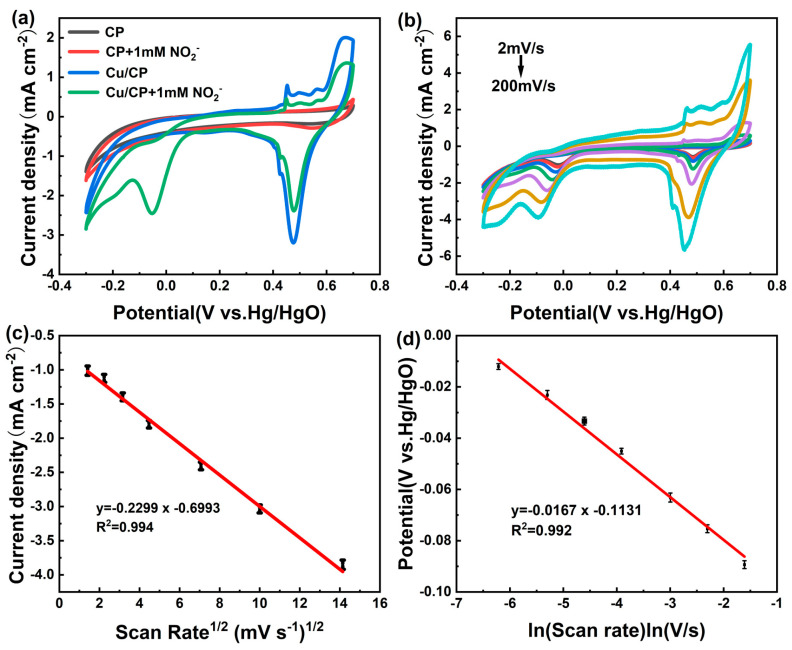
(**a**) CV curves of bare CP and Cu/CP−sensitive electrodes before and after addition of 1 mM nitrite in 0.1 M KOH electrolyte with a scan rate of 50 mVs^−1^. (**b**) CV curves of Cu/CP-sensitive electrodes in 0.1 M KOH solution containing 1 mM nitrite at different scan rates (2, 5, 10, 20, 50, 100, 200 mVs^−1^). (**c**) Linear relationship between reduction peak current and square root of scan rate. (**d**) Linear relationship between reduction peak potential and logarithm of scan rate.

To evaluate the impact of incorporating metallic Cu nanosheets onto the CP surface on the electrocatalytic reduction of nitrite, cyclic voltammetry (CV) was employed to compare the CV curves of the bare CP electrode and the Cu/CP-sensitive electrode before and after the introduction of nitrite, as illustrated in Figure 6a. In the absence of nitrite, no significant reduction peak was observed in the potential range around −0.05 V (vs. Hg/HgO) for any of the electrodes. Upon the addition of 1 mM nitrite, the Cu/CP-sensitive electrode exhibited a reduction peak around −0.05 V (vs. Hg/HgO), while the bare CP electrode displayed no notable change. This observation suggests that the Cu nanosheets possess exceptional catalytic capability for nitrite reduction. Furthermore, no corresponding oxidation peaks were detected in the CV curves of the electrodes, indicating the irreversible nature of the electrocatalytic reduction of nitrite.

To delve into the kinetics and mechanisms of the Cu/CP-sensitive electrode’s response to nitrite, CV curves were recorded for the electrode in 0.1 M KOH solution containing 1 mM nitrite at various scan rates, as shown in Figure 6b–d. The peak reduction current density (*I_pc_*) exhibited a linear relationship with the square root of the scanning rate (*v*^1/2^), as described by the linear regression equation: *I_pc_* (mA cm^−2^) = −0.2299*v*^1/2^ (mV/s) − 0.6993 (*R*^2^ = 0.994) (Figure 6c). This behavior suggests that the nitrite electrocatalytic reduction at the Cu/CP-sensitive electrode follows a diffusion-controlled process [29]. Additionally, a linear correlation between the reduction peak potential (*E_pc_*) and the logarithm of the scan rate (ln *v*) was observed, represented by the equation: *E_pc_* (V) = −0.0167ln *v* (ln V/s) − 0.1131 (*R*^2^ = 0.992) (Figure 6d). By fitting this relationship with the Laviron theoretical model, as stated in Equation (2) [30].

(2) $$E_{pc}=E^{\theta }+(\frac{RT}{\alpha nF})\ln(\frac{RTk}{\alpha nF})-\frac{RT}{\alpha nF}\ln\upsilon$$

In Equation (2), *E^θ^* is the standard electrode potential (V), *α* is the electron transfer coefficient, *k* is the standard rate constant (s^−1^) for the electrochemical oxidation of nitrite, and *T*, *R*, and *F* are the absolute temperature (298.15 K), the universal gas constant (8.314 J/(K⋅mol)), and Faraday’s constant (96,485 C/mol), respectively [31]. The number of transferred electrons (*n*) involved in the rate-determining step of the electrocatalytic reduction of nitrite at the Cu/CP-sensitive electrode was calculated to be about 3, considering an electron transfer coefficient *α* of 0.5 in a completely irreversible electrode reaction [32].

Based on the aforementioned results, the mechanism of the electrocatalytic reduction of nitrite at the Cu/CP-sensitive electrode can be inferred through Equations (3) to (7) [19]. Initially, nitrite adsorbs onto the Cu/CP-sensitive electrode surface to form adsorbed nitrite ions, followed by the reduction of water to form stable adsorbed hydrogen (H_ads_) upon passing current. In an alkaline environment, the adsorbed nitrite ions are progressively reduced by the adsorbed hydrogen to gain electrons, resulting in the formation of NO_ads_, N_2_O_ads_, and ultimately, the production of the stable compound N_2_ through a process involving a total of three electrons [19].

NO_2_^−^ → NO_2_^−^_ads_ (3)

H_2_O + e^−^ → H_ads_ + OH^−^ (4)

NO_2_^−^_ads_ + H_2_O + e^−^ → NO_ads_ + 2OH^−^ (5)

NO_ads_ + NO_sol_ + H_2_O + e^−^ → N_2_O_ads_ + 2OH^−^ (6)

N_2_O_ads_ + H_2_O + e^−^ → N_2_ + 2OH^−^ (7)

### 3.4. Sensitive Determination of Nitrite

**Figure 7:**
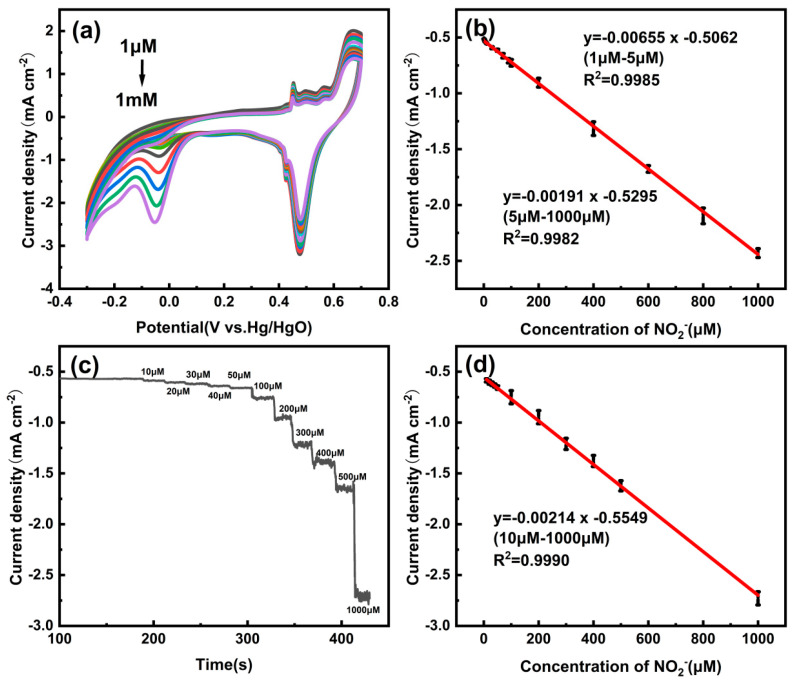
(**a**) CV curves of Cu/CP−sensitive electrodes in 0.1 M KOH electrolyte at a scan rate of 50 mVs^−1^ with different concentrations (1, 2, 3, 4, 5, 10, 20, 50, 100, 200, 400, 600, 800, 1000 μM) of nitrite added. (**b**) Linear relationship between reduction peak current and nitrite concentration. (**c**) Amperometric response of Cu/CP-sensitive electrodes to nitrite in 0.1 M KOH electrolyte at −0.05 V (vs. Hg/HgO) (**d**) Corresponding linear relationship between nitrite concentration and current response.

The linear detection of nitrite at the Cu/CP-sensitive electrode was successfully achieved through cyclic voltammetry, as illustrated in Figure 7a,b. The CV curves of the Cu/CP-sensitive electrode in 0.1 M KOH electrolyte with varying concentrations of nitrite (Figure 7a) demonstrate that the reduction peak current response magnitude increases with the introduction of nitrite in a gradual upward trend. This behavior indicates the diffusion of nitrite to the surface of the Cu/CP-sensitive electrode, where it undergoes reduction to facilitate electron transfer. These results can be attributed to the exceptional electrocatalytic activity, large electrochemically active surface area, and efficient electron transfer capability of the Cu/CP-sensitive electrode.

Figure 7b depicts the linear relationship between the reduction peak current density of the sensitive electrode and nitrite concentration. In the low concentration range of 1 to 5 μM, a strong linear correlation between the current response and nitrite concentration was observed: I_pc_ (mA cm^−2^) = −0.00655c (μM) − 0.5062 (R^2^ = 0.9985), with a sensitivity of 6500 μA mM^−1^cm^−2^. In the higher concentration range of 5 to 1000 μM, the current response exhibited a good linear relationship with nitrite concentration: I_pc_ (mA cm^−2^) = −0.00191c (μM) − 0.5295 (R^2^ = 0.9982), and the sensitivity in this range was 1910 μA mM^−1^cm^−2^. The limit of detection (LOD) for nitrite using the Cu/CP-sensitive electrode was exceptionally low at 0.083 μM, surpassing the WHO-recommended threshold of 65.2 μM for nitrite detection [33].

To achieve low-potential nitrite detection with the Cu/CP-sensitive electrode, a constant potential of −0.05 V (vs. Hg/HgO) was selected for linear nitrite detection using the timed current method, as depicted in Figure 7c,d. The results demonstrated a strong linear relationship between current response and nitrite concentration in the low concentration range of 10 to 1000 μM: I_pc_ (mA cm^−2^) = −0.00214c (μM) − 0.5549 (R^2^ = 0.9990), with a sensitivity of 2140 μA mM^−1^cm^−2^. The LOD for nitrite detection using this method was as low as 0.079 μM (S/N = 3), significantly below the WHO-recommended threshold. Comparison with other nitrite-sensitive electrodes reported previously (as shown in Table 1) highlights the exceptional performance of the Cu/CP-sensitive electrode in terms of low detection potential, LOD, sensitivity, and more, indicating its promising potential for quantitative nitrite detection.

In conclusion, the excellent electrochemical detection performance of the Cu/CP sensor for nitrite can be attributed to several key factors. The use of highly conductive carbon paper as the substrate ensures electrode conductivity and efficient electron transfer. The direct growth of Cu nanosheets on the carbon paper substrate eliminates the need for additional conductive polymer binders, reducing interfacial charge transfer impedance, enhancing electron transfer kinetics, and improving the current response for nitrite reduction. Furthermore, the Cu nanosheets exhibit superior catalytic properties for nitrite reduction, lowering the detection potential and providing a larger electrochemically active surface area with abundant catalytic sites, thereby enhancing the current response and sensitivity. The synergistic effects of these advantages contribute to the outstanding electrochemical detection performance of the Cu/CP electrode for nitrite.

### 3.5. Anti-Interference, Stability and Reproducibility of Cu/CP

**Figure 8:**
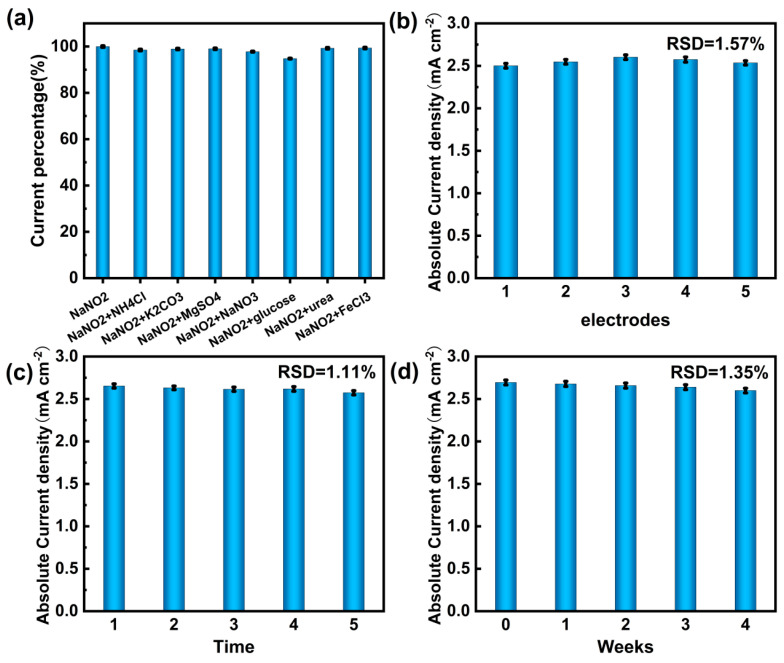
(**a**) Relative current percentage of Cu/CP−sensitive electrodes in 0.1 M KOH electrolyte with 1 mM nitrite and 1 mM interfering ions. (**b**) Comparative histogram of reduction peak currents of the same Cu/CP electrode for 5 consecutive tests with 1 mM nitrite. (**c**) Comparative histogram of reduction peak currents of 5 separate Cu/CP electrodes for 1 mM nitrite. (**d**) Histogram of peak currents of Cu/CP electrodes after 0–4 weeks of storage in 0.1 M KOH in the presence of 1 mM nitrite.

To assess the Cu/CP-sensitive electrode’s resilience to environmental complexity, its immunity to interference was scrutinized. Various interfering ions (NH_4_^+^, K^+^, Mg^2+^, Fe^3+^, SO_4_^2−^, CO_3_^2−^, Cl^−^, NO_3_^−^, urea, and glucose) at a concentration of 1 mM were introduced into a 0.1 M KOH electrolyte containing 1 mM nitrite. The selectivity and interference immunity of the Cu/CP electrodes were evaluated, with results depicted in Figure 8a. Notably, the current percentage histogram illustrated a minimal impact of the interfering ions on nitrite detection by the electrode, affirming the Cu/CP-sensitive electrode’s robust anti-interference performance.

The stability of the Cu/CP-sensitive electrode was assessed by subjecting the same electrode to cyclic voltammetry testing with 1 mM nitrite for five consecutive runs, as shown in Figure 8b. The results revealed consistent and nearly unchanged peak reduction currents, with a low relative standard deviation (RSD) of 1.11%, underscoring the excellent stability of the Cu/CP-sensitive electrode. Furthermore, cyclic voltammetry analysis of five electrodes prepared under identical conditions and their response to 1 mM nitrite (Figure 8c) yielded an RSD of 1.57%, indicating exceptional reproducibility of the Cu/CP-sensitive electrode.

Long-term stability was evaluated by monitoring the current response of the Cu/CP-sensitive electrode to 1 mM nitrite at weekly intervals over a 4-week period using cyclic voltammetry (Figure 8d). The results demonstrated consistent peak current responses over the 4-week duration, with an RSD of 1.35%. This consistency highlights the Cu/CP-sensitive electrode’s robust immunity to interference, reproducibility, and long-term stability. These characteristics ensure the sustainability and reliability of the electrochemical sensor for nitrite constructed with the Cu/CP-sensitive electrode in practical applications.

### 3.6. Actual Sample Testing

The practical applicability of Cu/CP-sensitive electrodes for nitrite detection in drinking water was demonstrated through the standard addition method. No pretreatment was required for the drinking water, allowing for direct quantitative determination. Each assay was replicated three times to ensure the reliability of the findings. As presented in Table 2, the recoveries ranged from 96.2% to 101.6%, with a relative standard deviation (RSD) of less than 1.89%. These results suggest that the Cu/CP-sensitive electrode is capable of detecting nitrite in real-world samples.

## 4. Conclusions

Self-supporting nitrite electrochemical sensors were fabricated by utilizing a simple one-step constant potential electrodeposition technique to grow copper nanosheets directly on carbon paper substrates. The combination of the catalytic efficiency of copper and the high electrical conductivity of carbon paper facilitated the development of Cu/CP-sensitive electrodes with large electrochemically active surface areas and fast electron transfer rates. This resulted in impressive nitrite sensing capabilities, including a wide linear detection range (10–1000 μM), low detection limit (0.079 μM at S/N = 3), low reduction detection potential (−0.05 V vs. Hg/HgO), high sensitivity (2140 μA mM^−1^cm^−2^), and excellent immunity to interference and stability, as well as excellent practical detection capabilities. In summary, this work provides a reference for advancing electrochemical low-potential nitrite detection methods.

## Figures and Tables

**Table 1:** Comparison of Cu/CP with other nitrite sensors.

Electrode Materials	Sensitivity (μA mM^−1^cm^−2^)	Linear Range (μM)	LOD (μM)	Detection Potential (V)	Reference
Cu@CeO2-rGO	1963.2	10–2000	0.0101	0.8 V (vs. SCE)	[34]
Cu-Co oxide NPs	74.36	100–2800	0.5	0.75 V (vs. Ag/AgCl)	[35]
Ag/Cu/MWNTs/GCE	380.9	1–1000	0.2	0.85 V (vs. Ag/AgCl)	[36]
CuO NPs/CC	1656	0.5–3000	0.043	0.8 V (vs. SCE)	[15]
CuO/MWCNTs/SPE	501	0.1–6500	0.039	0.85 V (vs. Ag/AgCl)	[37]
CuO/NiO/FTO	496.6	1–1800	0.013	1 V (vs. SCE)	[38]
Cu-MOF/Au	17	0.1–4000	0.082	0.8 V (vs. SCE)	[39]
PANI-NF	845	0.2–3500	0.05	0.4 V (vs. SCE)	[17]
Hb/PLE	2047	10–220	5	−1.75 V (vs. Ag/AgCl)	[40]
Cu/CP	2140	10–1000	0.079	−0.05 V (vs. Hg/HgO)	This work

**Table 2:** Electrochemical detection of nitrite in drinking water.

Actual Samples	Initial (μM)	Added (μM)	Found (μM)	Recovery (%)	RSD (%, n = 3)
Drinking water	0	10	9.87	98.7	1.06
		20	19.24	96.2	1.44
		50	50.78	101.6	1.89

## Data Availability

The data that has been used is confidential.
